# Disparities in Utilization and Outcomes of Minimally Invasive Techniques for Gastric Cancer Surgery in the United States

**DOI:** 10.1245/s10434-021-11193-6

**Published:** 2022-01-07

**Authors:** Joon Y. Park, Arjun Verma, Zachary K. Tran, Michael A. Mederos, Peyman Benharash, Mark Girgis

**Affiliations:** Department of Surgery, David Geffen School of Medicine, UCLA Surg-Surg Onc, Los Angeles, CA USA

## Abstract

**Background:**

This study investigated national implementation patterns and perioperative outcomes of minimally invasive gastrectomy (MIG) in gastric cancer surgery in the United States.

**Methods:**

The National Inpatient Sample (NIS) was queried for patients who underwent elective gastrectomy for gastric cancer from 2008-2018. The MIG versus open gastrectomy approach was correlated with hospital factors, patient characteristics, and complications.

**Results:**

There was more than a fivefold increase in MIG from 5.8% in 2008 to 32.9% in 2018 (nptrend < 0.001). Patients undergoing MIG had a lower Elixhauser Comorbidity Index (*p* = 0.001). On risk adjusted analysis, black patients (AOR = 0.77, *p* = 0.024) and patients with income below 25^th^ percentile (AOR = 0.80, *p* = 0.018) were less likely to undergo MIG. When these analyses were limited to minimally invasive capable centers only, these differences were not observed. Hospitals in the upper tertile of gastrectomy case volume, Northeast, and urban teaching centers were more likely to perform MIG. Overall, MIG was associated with a 0.7-day decrease in length of stay, reduced risk adjusted mortality rates (AOR = 0.58, *p* = 0.05), and a $4,700 increase in total cost.

**Conclusions:**

In this national retrospective study, we observe socioeconomic differences in patients undergoing MIG, which is explained by hospital level factors in MIG utilization. We demonstrate that MIG is associated with a lower mortality compared with open gastrectomy. Establishing MIG as a safe approach to gastric cancers and understanding regional differences in implementation patterns can inform delivery of equitable high-quality health care.

**Supplementary Information:**

The online version contains supplementary material available at 10.1245/s10434-021-11193-6.

Gastric cancer is the fourth leading cause of cancer-related mortality worldwide.^1^ Despite advances in systemic therapy, surgical resection remains the only curative treatment. Although minimally invasive approaches were introduced in 1994, the open approach has remained the preferred surgical approach for resectable gastric cancers.^2^ In the early 2010s, a series of randomized phase III control trials conducted in East Asian countries established the superiority of the minimally invasive gastrectomy (MIG) compared to open surgery in early gastric cancer, citing improved length of stay, blood loss and complications rates.^3,4^ Follow-up data demonstrated equivalent long-term oncologic outcomes between open, laparoscopic and robotic gastrectomy.^5–7^ Randomized trials applying minimally invasive approaches to advanced gastric cancers, which are traditionally more complex, are currently underway with early results demonstrating similar short-term outcomes.^8–11^

Importantly, the East Asian population differs significantly from the Western population in several respects. Due to the high incidence of gastric cancer in these countries, there are rigorous screening guidelines for gastric cancer, resulting in more frequent presentation of early (i.e., resectable) disease. In addition, the Western population presents more frequently with proximal and diffuse histologic subtypes, which require total gastrectomy and carry a worse overall prognosis.^12^ Thus, the utility of MIG in the United States is yet to be well characterized.

Despite the lack of randomized control trials supporting MIG in the United States, the rate of MIG is increasing. Several studies have attempted to define outcomes following MIG with inconclusive results. A trend toward improved overall survival with MIG was observed in a combined Western and Eastern retrospective study.^13^ Most recently, two studies utilizing the National Cancer Database (NCDB) analyzing survival following MIG presented opposing conclusions despite using the same time period with one study finding improved short-term and long-term survival with MIG.^14,15^ These studies also had conflicting findings regarding socioeconomic utilization of MIG. Notably, as the NCDB only includes data from hospitals approved by the committee on cancer, it is thought to inherently bias the database toward better oncologic outcomes and underrepresentation of socioeconomic disparities.^16–18^ Conversely, the National Inpatient Sample (NIS), an all-payer database that estimates >97% of inpatient stays in the United States, better captures national trends in procedures, demographics, and outcomes. The NIS also includes information about total costs associated with hospitalization not available in the NCDB.

Using the NIS, we sought to investigate the utilization of the MIG in the United States and to understand whether the perioperative benefits of MIG is replicated in a Western population.^5–7^ We hypothesized that there would be geographic and socioeconomic differences in MIG implementation and that MIG would have superior short-term outcomes compared with open gastrectomy.

## Methods

This was a retrospective cohort study of the 2008-2018 National Inpatient Sample (NIS). The study period marks the transition point between the establishment of MIG as a safe approach to gastric cancers with landmark studies.^5–7^ The NIS is the largest national all-payer inpatient database in the United States. Before 2012, the NIS was constructed on 100% of discharge records from 20% of hospitals.^19^ Starting in 2012, the NIS began to sample 20% of discharges from all participating hospitals. Validated sampling algorithms are used to provide accurate estimates for 97% of all United States hospitalizations. *International Classification of Diseases, 9*^*th*^* and 10*^*th*^* Edition* (ICD-9, ICD-10) diagnosis and procedure codes were used to identify all adult patients who underwent elective gastrectomy for gastric adenocarcinoma (Supplementary). Those with trauma-related admission, benign gastric masses or disease, and other gastric tumor subtypes, such as gastrointestinal stromal tumors and carcinoids, were excluded from analysis. Patients with missing data for age, sex, mortality, and hospitalization cost data also were excluded. Patients were stratified by surgical approach into open, laparoscopic, and robot-assisted using ICD-9/10 procedure codes. Those who underwent open procedures comprised the *Open* cohort, while laparoscopic and robot-assisted procedures were grouped as *MIG*.

Patient and hospital characteristics, including age, sex, race, income level, payer status, hospital teaching status, and region, were defined in accordance with the Healthcare Cost and Utilization Project data dictionary.^20^ The van Walraven modification of the Elixhauser Comorbidity Index was used to numerically tabulate the extent of chronic conditions.^21^ Patients also were stratified by extent of resection (partial vs. total gastrectomy) as well as by those undergoing concomitant operations (splenectomy, colectomy, feeding jejunostomy, or pancreatectomy). Hospitals were stratified into low-, medium-, and high-volume tertiles based on annual institutional caseload of gastrectomy for gastric cancer using previously validated methodology.^22^ As the NIS does not track hospitals across years, MIG capable centers were defined as those who performed at least one minimally invasive surgery for gastric cancer in each calendar year. Complications also were identified using ICD-9/10 procedure codes and were grouped into cardiac (ventricular tachycardia, ventricular fibrillation, cardiac arrest, cardiac tamponade), thrombotic (deep vein thrombosis, pulmonary embolism), respiratory (pneumonia, empyema, invasive mechanical ventilation >96 hours, pneumothorax, respiratory failure), and infectious (urinary tract infection, bacterial infection, sepsis, infectious postoperative seroma, wound disruption, *Clostridium difficile* colitis, peritoneal abscess) categories. Hospitalization costs were defined by application of hospital-specific, cost-to-charge ratios to overall charges, and inflation was adjusted to the 2018 Personal Health Care Index.^23^

The primary outcome of interest was the utilization patterns of open gastrectomy, laparoscopic gastrectomy, and robotic gastrectomy. Secondary outcomes of interest included in-hospital mortality, complications, length of stay (LOS), and hospitalization costs.

Statistical analysis was performed using Stata 16.0 (StataCorp, College Station, TX) software. Temporal trends were analyzed using a rank-based, nonparametric test by Cuzick (nptrend) (Cuzick, 1985). Differences in temporal trends by testing for interaction between groups in a multiple linear regression model. Categorical variables are reported as proportions (%) and were analyzed using the Pearson’s chi-square test. Continuous variables are reported as means with standard deviations (SD) and were compared using an adjusted Wald test. Logistic regression models were developed to identify patient, operative, and hospital characteristics associated with the utilization of MIG. Additional models were developed to explore the risk-adjusted impact of MIG on in-hospital mortality, complications, LOS, and hospitalization costs. Regression outcomes are reported as adjusted odds ratios (AOR) for discrete or ß-coefficients for continuous variables, both with 95% confidence intervals (95% CI). Elastic net regression—a machine-learning technique that combines LASSO and ridge regularization—was utilized for variable selection to develop a model with minimal collinearity and optimal discrimination. Following retention of clinically relevant variables, the final models were optimized using the area under the receiver operating characteristic (C-Statistic), as well as the Akaike and Bayesian Information Criterion. Statistical significance was set at α < 0.05. This study was deemed exempt from full review by the Institutional Review Board at the University of California, Los Angeles.

## Results

### MIG Utilization Over Time

**Fig. 1:**
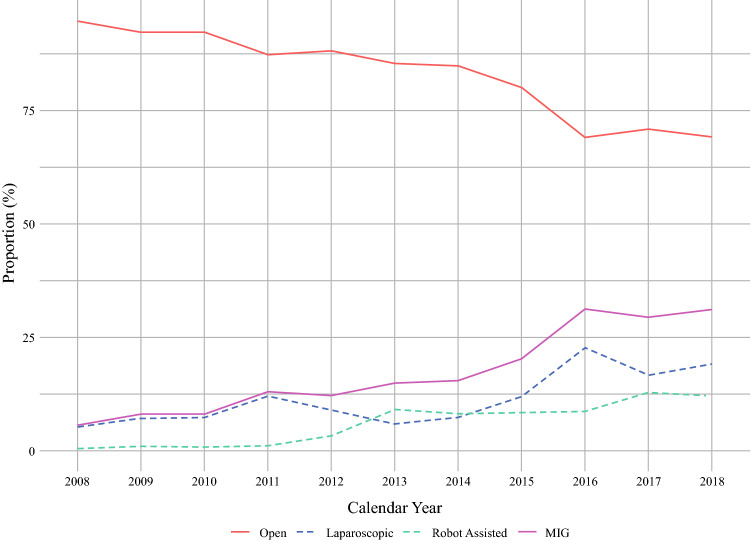
Trends in gastrectomy approach for gastric cancer

**Fig. 2:**
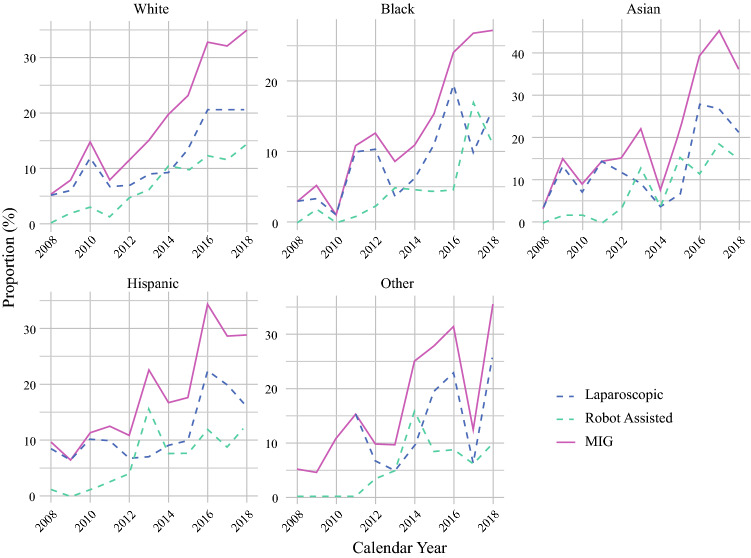
Trends in MIG utilization by race

Of an estimated 41,758 hospitalizations for gastrectomy during the study period, 7,242 (17.3%) were considered *MIG*. Utilization of MIG increased significantly over time, from 5.8% of all gastrectomies in 2008 to 32.9% in 2018 (nptrend < 0.001). Robotic procedures had a 60-fold increase from 0.2% of all gastrectomies in 2008 to 13.0% in 2018, whereas the laparoscopic approach had a 3-fold increase from 5.6% in 2008 to 19.8% in 2018 (Fig. 1, nptrend < 0.001). White patients had the highest proportion of *MIG* utilization at the beginning of the study period at 5.4%, whereas black patients had the lowest proportion at 3.0% (Fig. 2). There were no significant differences in trends of MIG utilization by race over time.

### Baseline Characteristics of Open Gastrectomy versus Minimally Invasive Gastrectomy

The *Open* and *MIG* cohorts were similar in age and sex (Table 1). Total gastrectomies made up a larger proportion of the *Open* group (*Open*: 33.9% vs. *MIG*: 26.7%, *p* < 0.001). Gastrectomies that involved a concomitant splenectomy, colectomy, or pancreatectomy were more often done via the *Open* approach (Table 1). Patients in the *Open* group on average had a marginally higher Elixhauser comorbidity index (*Open:* 3.60 ± 1.68 vs. *MIG:* 3.45 ± 1.62, *p* = 0.004). Specifically, those with congestive heart failure, history of weight loss, electrolyte disorders, and metastatic disease more frequently received *Open* (Table 1).

**Table 1:** Baseline clinicopathologic, socioeconomic, and hospital factors patients undergoing open and minimally invasive gastrectomy

Parameter	Open (34,516)	MIG (7,242)	*p* value
*Clinicopathologic factors*			
Age (yr, mean ± SD)	65.2±12.4	65.0±12.3	0.46
Female	32.8%	34.7%	0.16
*Gastrectomy type*			
Partial	66.1%	73.3%	**< 0.001**
Total	33.9%	26.7%	
*Concomitant operation*			
Splenectomy	4.3%	2.5%	**0.003**
Colectomy	1.8%	0.8%	**0.004**
Feeding jejunostomy	38.6%	36.4%	0.20
Pancreatectomy	2.1%	1.1%	**0.013**
Elixhauser Comorbidity Index (mean ± SD)	3.60 ± 1.68	3.45 ± 1.62	**0.004**
*Medical conditions*			
Congestive heart failure	6.0%	3.8%	**0.001**
Coronary artery disease	15.8%	15.4%	0.67
Arrhythmia	24.9%	24.3%	0.67
Valve disorder	3.6%	3.9%	0.56
Pulmonary circulatory disorder	2.3%	2.0%	0.49
Peripheral vascular disease	3.6%	4.1%	0.42
Hypertension	53.2%	53.7%	0.74
Neurologic disorder	3.7%	3.1%	0.31
Chronic lung disorder	15.9%	14.7%	0.26
Diabetes	22.8%	20.9%	0.10
Hypothyroidism	8.8%	9.5%	0.40
End stage renal disease	1.0%	0.7%	0.31
Liver disease	5.8%	6.0%	0.79
Peptic ulcer disease	2.9%	3.4%	0.30
Metastatic cancer	30.6%	26.5%	**0.005**
Coagulopathy	4.4%	5.6%	0.058
Weight Loss	17.4%	14.5%	**0.013**
Electrolyte disorder	25.1%	18.6%	**< 0.001**
Anemia	6.6%	5.7%	0.23
*Socioeconomic factors*			
*Race*			
White	57.9%	60.1%	0.23
Black	10.9%	8.5%	**0.007**
Hispanic	10.3%	11.2%	0.38
Asian/Pacific Islander	7.2%	9.7%	**0.007**
Other	4.1%	4.2%	0.84
*Income (Percentile)*			
76th-100^th^	25.9%	31.5%	**< 0.001**
51st-75^th^	24.0%	23.8%	0.87
26th-50^th^	23.7%	24.5%	0.54
0th-25^th^	24.5%	18.7%	**< 0.001**
*Payer status*			
Private	36.5%	36.6%	0.94
Medicare	51.7%	50.9%	0.59
Medicaid	7.5%	8.4%	0.25
Other payer	4.2%	3.8%	0.48
*Hospital characteristics*			
*Region*			
Northeast	20.4%	34.3%	**< 0.001**
Midwest	22.3%	16.9%	**< 0.001**
South	37.6%	28.2%	**< 0.001**
West	19.6%	20.5%	0.62
*Teaching status*			
Rural	2.9%	1.4%	**< 0.001**
Urban nonteaching	20.9%	9.6%	**< 0.001**
Urban teaching	75.7%	88.9%	**< 0.001**
*Gastrectomy volume (Percentile)*			
0th-33rd	14.3%	9.9%	**< 0.001**
34th-66th	16.3%	10.6%	**< 0.001**
67th-100th	69.4%	79.5%	**< 0.001**

Although the *Open* and *MIG* cohorts had similar insurance payer status, the *MIG* group had a higher proportion of patients in the highest quartile of income (*Open* 25.9% vs. *MIG* 31.5%, *p* < 0.001) and a lower proportion of patients in the lowest quartile of income (*Open* 24.5% vs. *MIG* 18.7%, *p* < 0.001). Additionally, black patients more frequently underwent *Open* (*Open* 10.9% vs. *MIG* 8.5%, *p* = 0.007), whereas Asian/Pacific Islanders were more often received *MIG* (*Open* 7.2% vs. *MIG* 9.7%, *p* = 0.007). The distribution of all other income quartiles and race categories were similar between the *Open* and *MIG* groups.

Hospitals in the Northeast more frequently performed *MIG* (*Open* 20.4% vs. *MIG* 34.3%, *p* < 0.001), whereas hospitals in the Southwest or West more frequently performed *Open* gastrectomy (Table 1). As a whole, urban teaching hospitals accounted for the majority of all gastrectomies and also more frequently performed *MIG* than urban-nonteaching or rural hospitals (*p* < 0.001). Similarly, the *MIG* cohort had a higher proportion of hospitals in the upper tertile of gastrectomy volume than the middle or lower tertiles (*p* < 0.001).

### Risk Adjusted Independent Factors Associated with MIG Utilization

In the risk-adjusted model, independent clinicopathologic factors associated with a higher likelihood of *Open* were patients with total gastrectomy (AOR 0.79 *p* = 0.001), congestive heart failure (AOR 0.68 *p* = 0.02), metastatic cancer (AOR 0.86 *p* = 0.04.), history of weight loss (AOR 0.82 *p* = 0.03), and electrolyte disorders (AOR 0.72 *p* < 0.001). Socioeconomic predictors of *Open* included Black patients (AOR 0.77 *p* = 0.024) and patients in the lowest income quartile (AOR 0.80 *p* = 0.018). Finally, hospital factors associated with higher likelihood of *MIG* were hospitals in the Northeast and those in the highest tertile of gastrectomy volume (Table 2).

**Table 2:** Risk-adjusted factors associated with utilization of minimally invasive gastrectomy in all centers and MIG capable centers only

Parameter	All centers		MIG capable centers	
	AOR (95 CI)	*p* value	AOR (95 CI)	*p* value
*Clinicopathologic factors*				
Age (per-year)	1.00 (1.00-1.01)	0.30	1.00 (1.00-1.01)	0.44
Female sex	1.07 (0.94-1.23)	0.30	1.17 (0.99-1.37)	0.058
Year of operation (per-year)	1.24 (1.21-1.26)	**< 0.001**	1.26 (1.23-1.29)	**< 0.001**
*Procedure type*				
Partial	Ref		Ref	
Total	0.79 (0.68-0.91)	**0.001**	0.82 (0.70-0.97)	**0.023**
*Concomitant operation*				
Splenectomy	0.86 (0.56-1.30)	0.48	1.06 (0.65-1.73)	0.82
Feeding jejunostomy	1.14 (0.99-1.31)	0.069	1.04 (0.88-1.22)	0.65
Colectomy	0.47 (0.24-0.92)	**0.027**	0.44 (0.20-0.96)	**0.039**
Pancreatectomy	0.51(0.27-0.96)	**0.038**	0.63 (0.30-1.31)	0.22
*Medical conditions*				
Congestive heart failure	0.68 (0.50-0.93)	**0.015**	0.66 (0.45-0.95)	**0.027**
Valve disorder	1.31 (0.94-1.82)	0.11	1.44 (0.96-2.14)	0.076
Pulmonary circulatory Disorder	0.91 (0.59-1.41)	0.68	0.83 (0.51-1.36)	0.46
Hypertension	1.00 (0.87-1.14)	0.97	1.01 (0.86-1.18)	0.93
Neurologic disorder	0.88 (0.62-1.27)	0.50	1.23 (0.80-1.89)	0.34
Chronic lung disorder	1.02 (0.85-1.22)	0.84	1.02 (0.82-1.26)	0.88
Diabetes	0.86 (0.73-1.01)	0.067	0.86 (0.71-1.03)	0.11
Hypothyroidism	0.93 (0.75-1.16)	0.53	0.85 (0.65-1.11)	0.22
End-stage renal disease	0.87 (0.75-1.16)	0.70	0.96 (0.43-2.13)	0.92
Metastatic cancer	0.86 (0.75-0.99)	**0.040**	0.88 (0.75-1.04)	0.13
Coagulopathy	1.25 (0.94-1.66)	0.12	1.23 (0.88-1.72)	0.22
Weight loss	0.82 (0.69-0.98)	**0.032**	0.81 (0.66-0.99)	**0.047**
Electrolyte disorder	0.72 (0.61-0.84)	**< 0.001**	0.77 (0.64-0.93)	**0.006**
*Socioeconomic factors*				
*Payer status*				
Private	Ref		Ref	
Medicare	1.03 (0.86-1.23)	0.74	1.01 (0.83-1.24)	0.91
Medicaid	1.06 (0.83-1.36)	0.65	1.11 (0.83-1.48)	0.59
Other payer	0.91 (0.65-1.29)	0.61	0.78(0.52-1.17)	0.23
*Race*				
White	Ref		Ref	
Black	0.77 (0.61-0.97)	**0.024**	0.78 (0.60-1.03)	0.080
Hispanic	1.04 (0.84-1.28)	0.75	1.08 (0.84-1.39)	0.54
Asian/Pacific Islander	1.01 (0.81-1.27)	0.91	1.04 (0.79-1.36)	0.79
Other	0.87 (0.64-1.19)	0.39	0.90 (0.63-1.29)	0.57
*Income quartile (percentile)*				
76th-100th	Ref		Ref	
51st-75th	0.93 (0.78-1.10)	0.41	0.95 (0.77-1.16)	0.59
26th-50th	1.08 (0.91-1.29)	0.38	1.16 (0.94-1.42)	0.16
0th-25th	0.80 (0.66-0.96)	**0.018**	0.90 (0.72-1.12)	0.33
*Hospital factors*				
Hospital region				
Northeast	Ref		Ref	
Midwest	0.54 (0.44-0.66)	**< 0.001**	0.77 (0.61-0.97)	**0.028**
South	0.54 (0.45-0.63)	**< 0.001**	0.90 (0.74-1.09)	0.27
West	0.75 (0.62-0.90)	**0.002**	0.94 (0.76-1.16)	0.55
*Hospital teaching status*				
Rural	Ref		Ref	
Urban nonteaching	0.96 (0.55-1.67)	0.89	1.19(0.60-2.36)	0.62
Urban teaching	1.59 (0.94-2.71)	0.085	1.06 (0.55-2.04)	0.86
*Gastrectomy volume tertile*				
Low	Ref		Ref	
Medium	1.13 (0.86-1.47)	0.39	1.02 (0.71-1.47)	0.91
High	1.62 (1.32-2.01)	**< 0.001**	0.81 (0.61-1.09)	0.16

**Fig. 3:**
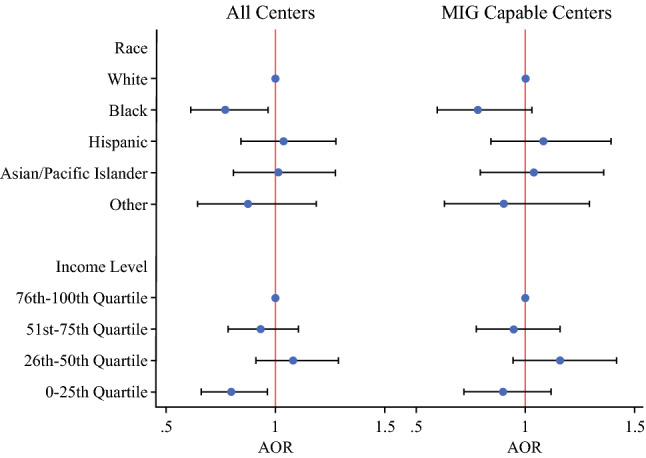
Socioeconomic disparities in access to minimally invasive gastrectomy

There were several differences when this model was limited to data from *MIG*-capable centers only. Notably, there were no identified socioeconomic factors that were associated with *Open* versus *MIG* (Fig. 3). Other than hospitals in the Midwest, which were more likely to perform *Open*, hospital level factors including location, gastrectomy volume, and teaching status were also not associated with *Open* versus *MIG*. Patients with total gastrectomy, colectomy, congestive heart failure, weight loss and electrolyte disorders were still more likely to undergo *Open* in this subanalysis (Table 2).

### Postoperative Outcomes Associated with MIG

The *Open* group had significantly higher rates of in-hospital mortality (*Open* 2.9% vs. *MIG* 1.4%, *p* = 0.003) and higher incidence of cardiac, respiratory, gastrointestinal, infectious, and acute kidney injury complications (Table 3). Thrombotic complications were similar between both cohorts. Rates of nonroutine discharge were similar between the two cohorts (*Open* 49.8% vs. *MIG* 47.4% *p* = 0.13). Patients in the *Open* cohort experienced a longer length of stay (LOS) but lower hospitalization costs (Table 3).

**Table 3:** Unadjusted and risk-adjusted outcomes of open and minimal access gastrectomy

Parameter	Open (34,516)	MIG (7,242)	*p* value	AOR/β (95% CI)	*p* value
Mortality	2.9%	1.4%	**< 0.001**	0.58 (0.34-1.00)	**0.050**
*Complications*					
Cardiac	2.1%	1.2%	**0.028**	0.72 (0.42-1.24)	0.23
Thrombotic	3.0%	2.1%	0.11	0.73 (0.44-1.22)	0.23
Respiratory	20.8%	18.3%	**0.040**	1.04 (0.88-1.23)	0.64
Gastrointestinal	3.8%	5.2%	**0.026**	0.89 (0.66-1.22)	0.48
Infectious	11.9%	10.0%	**0.038**	0.90 (0.73-1.11)	0.33
Acute kidney Injury	7.7%	6.0%	**0.040**	0.93 (0.70-1.22)	0.59
Nonroutine discharge	49.8%	47.4%	0.13	0.91 (0.78-1.06)	0.22
LOS (days, mean ± SD)	11.9 ± 10.5	9.9 ± 9.6	**< 0.001**	−0.7 (−1.3- −0.2)	**0.011**
Cost ($1,000, mean ± SD)	39.9 ± 42.0	42.3 ± 41.8	0.064	4.7 (2.2-7.1)	**< 0.001**

Multivariable regression results reported as adjusted odds ratios (AOR) or β-coefficients for discrete and continuous variables, respectively

*CI* confidence interval; *LOS* length of stay; *MIS* minimal access gastrectomy; *SD* standard deviation

When risk adjusted, *MIG* was independently associated with lower mortality odds of in-hospital mortality (AOR 0.58 *p* = 0.050), but not with specific perioperative complications or non-routine discharge (Table 3). *MIG* was associated with a 0.7-day decrement in LOS and a $4,900 increase in attributable hospital costs. Sub-analysis between laparoscopic and robotic assisted gastrectomy also did not demonstrate any differences in measured postoperative outcomes (Supplementary).

## Discussion

The present work represents the largest national analysis to characterize adoption patterns and short-term outcomes of minimally invasive gastrectomy for gastric cancer in the United States. Our data demonstrate increasing utilization of *MIG* over the past decade. While increasing comorbidities, black race and low income were associated with a lower likelihood of *MIG*, this association disappeared in hospitals that were capable of performing minimally invasive operations. Importantly, *MIG* appears associated with decrements in LOS and mortality but increases in overall costs.

It is widely accepted that minimally invasive approaches in common general surgery operations are associated with less morbidity, and racial inequality in accessing minimally invasive surgery has been previously observed.^24^ Such inequalities certainly contribute to known racial disparities in surgical outcomes, and it is important to understand the mechanisms that contribute to differences in application of MIG.^25–28^ Geographic biases in minimally invasive surgery have been previously shown and is thought to reflect regional training patterns.^29^ The increasing incidence of *MIG* in the Northeast seen in our data and observed previously is likely a reflection of the greater concentration of complex surgical oncologic training fellowships in this region.^14,30^ Similarly, the greater rate of MIG in urban teaching centers likely reflects the trend of “urbanization” of specialized general surgery and narrowing scope of practice of a rural surgeon.^31,32^

Because there was no significant difference in *MIG* utilization among black and low-income patients presenting to *MIG* capable centers, it is likely that unequal access to these hospitals drive the observed differences in *MIG* utilization in these populations. Centralization of gastric cancer care is supported by repeated studies demonstrating that patients who receive care at high-volume centers with experienced surgeons have less perioperative morbidity.^33–37^ The best example of this is in the Netherlands, when it was mandated in 2012 that gastric cancer operations be performed in high-volume centers, defined by greater than 20 gastric resections per year. A recent study analyzing the impact of this mandate confirmed not only decreased perioperative mortality, but also increased overall survival.^38^ Naturally, core discrepancies exist between the healthcare systems of Netherlands and the United States. For example, a recent NCDB Database study showed that gastric cancer patients that presented to medium- or high-volume centers traveled significantly further than those that present to low-volume centers and were more likely to be insured.^33^ Further centralization of complex surgical care may have the unintended consequence of limiting access for the most vulnerable populations unable to travel the distance or obtain insurance approval and increase the observed disparity in MIG utilization shown here. Instead, we advocate for the growth of the minimally invasive platform across the healthcare system via systematic training and adoption of new technology so that minimally invasive capable surgeons can populate hospitals in areas of underserved communities.

The perioperative outcomes presented here are largely consistent with randomized studies in East Asia comparing *Open* and *MIG* that have repeatedly demonstrated noninferior short-term outcomes in both total and distal gastrectomies.^3,4,39–41^ As previously discussed, there exists substantial differences between the East Asian and Western presentations of gastric cancers, resulting in the typical surgeon in the United States seeing less volume and more advanced cases. These differences are especially important as the learning curve of MIG is especially steep, requiring anywhere from 40 cases for distal gastrectomy to up to 100 cases for total gastrectomy.^42,43^ The additional technical complexity of the minimally invasive total gastrectomy explains the observed higher rates of the distal MIG compared with total gastrectomy. Despite this, among those undergoing MIG in the United States, rates of postoperative complications were equal between open and minimally invasive gastrectomy in this study. Interestingly, consistent with a previous NCDB study, we also observed a lower mortality rate in the MIG cohort, which is most likely explained by surgeon selection bias as evidenced by the lower comorbidity burden in this population.^14^ Additionally, despite that MIG was associated with a 0.7-day decrement in LOS, consistent with previous studies, MIG still had a $4,900 increase in total hospitalization cost over open surgery. This increase is most likely due to previously observed higher upfront cost of MIG which includes increased total operative time and surgical instrument costs, although operative time was not available in this dataset.^44–46^ Notably, these factors and consequent costs are especially exaggerated in robotic gastrectomy.^46,47^

This study has several important limitations. The dataset studied is limited to a single admission for patients undergoing gastrectomy. Thus, information before admission and additional follow-up data following discharge is not available. Importantly, this includes indication for surgery, such as oncologic resection versus symptom palliation. Physician-specific factors, such as surgical volume and MIG experience, were not available in this study. Additionally, the NIS relies on accurate administrative coding for outcome data and is subject to coding error. Finally, the retrospective nature of this study subjects it to inherent biases, such as patient selection.

## Conclusions

We have shown in a large national retrospective review that observed differences in MIG rates in Black and low-income populations are explained in part by inequalities in accessing MIG capable centers. We show that MIG is associated with a 0.7-day decreased length of stay, a $4,700 increase in total cost, and decreased mortality. Further research and thoughtfully designed randomized trials are required to identify modifiable factors to increase equitable access to MIG and establish MIG as a safe approach to gastric cancers.

## Supplementary Information

Below is the link to the electronic supplementary material.

Supplementary file1 (DOCX 21 kb)
