# Supplementary material for: Disparities in Utilization and Outcomes of Minimally Invasive Techniques for Gastric Cancer Surgery in the United States
Source: Ann Surg Oncol. 2022 Jan 7;29(5):3136–46. doi: 10.1245/s10434-021-11193-6 (PMC8990946; doi:10.1245/s10434-021-11193-6)
Supplement: Supplementary file 1 — Supplementary file1 (DOCX 21 kb) [file 10434_2021_11193_MOESM1_ESM.docx]

Supplemental Table 1: ICD codes queried in this study

| **Diagnosis/Procedure** | **ICD-9** | **ICD-10** |
| --- | --- | --- |
| Partial Gastrectomy | 435, 436, 437, 4381, 4382, 4389 | 0DB60, 0DB70, 0DB63, 0DB73, 0DB64, 0DB74, 0DB67, 0DB77, 0DB68, 0DB78 |
| Total Gastrectomy | 4391, 4399 | 0DT60, 0DT70, 0DT64, 0DT75, 0DT67, 0DT77, 0DT68, 0DT78 |
| Laparoscopic | 5451, 5421 | 0DB64ZZ, 0DB74ZZ. 0DT64ZZ, 0DT74ZZ |
| Robot Assisted | 1741, 1742, 1743, 1744, 1745, 1749 | 8E0W0CZ, 8E0W4CZ |

Supplemental Table 2: Baseline characteristics of patients undergoing laparoscopic gastrectomy and robotic gastrectomy

| **Parameter** | **Laparoscopic (4,698)** | **Robotic (2,544)** | **P-Value** |
| --- | --- | --- | --- |
| **Clinicopathologic Factors** |  |  |  |
| Age (years, mean±SD) | 65.1±12.7 | 64.8±11.9 | 0.65 |
| Female | 36.6% | 31.3% | **0.030** |
| Gastrectomy Type |  |  |  |
| Partial | 72.5% | 74.8% | 0.44 |
| Total | 27.5% | 25.2% |  |
| Concomitant Operation |  |  |  |
| Splenectomy | 3.0% | 1.6% | 0.10 |
| Colectomy | 0.8% | 0.8% | 0.97 |
| Feeding Jejunostomy | 34.4% | 39.8% | 0.092 |
| Pancreatectomy | 1.5% | 0.3% | 0.060 |
| Elixhauser Comorbidity Index (mean±SD) | 3.46±1.64 | 3.43±1.60 | 0.74 |
| Medical Conditions |  |  |  |
| Congestive Heart Failure | 3.3% | 4.7% | 0.20 |
| Coronary Artery Disease | 14.6% | 16.8% | 0.28 |
| Arrhythmia | 24.7% | 23.6% | 0.65 |
| Valve Disorder | 3.8% | 4.1% | 0.72 |
| Pulmonary Circulatory Disorder | 1.9% | 2.3% | 0.52 |
| Peripheral Vascular Disease | 4.0% | 4.3% | 0.76 |
| Hypertension | 54.4% | 52.4% | 0.47 |
| Neurologic Disorder | 3.1% | 3.1% | 0.97 |
| Chronic Lung Disorder | 14.7% | 14.6% | 0.93 |
| Diabetes | 21.2% | 20.4% | 0.72 |
| Hypothyroidism | 9.1% | 10.2% | 0.50 |
| Acute Kidney Injury | 0.5% | 1.0% | 0.26 |
| Liver Disease | 6.0% | 6.1% | 0.93 |
| Peptic Ulcer Disease | 3.5% | 3.3% | 0.89 |
| Metastatic Cancer | 25.5% | 28.2% | 0.34 |
| Coagulopathy | 5.8% | 5.3% | 0.71 |
| Weight Loss | 15.2% | 13.1% | 0.35 |
| Electrolyte Disorder | 19.2% | 17.4% | 0.46 |
| Anemia | 6.3% | 4.7% | 0.20 |
| **Socioeconomic Factors** |  |  |  |
| Race |  |  |  |
| White | 59.1% | 61.8% | 0.43 |
| Black | 8.7% | 8.0% | 0.71 |
| Hispanic | 10.9% | 11.6% | 0.71 |
| Asian/Pacific Islander | 9.4% | 10.2% | 0.71 |
| Other | 4.5% | 3.7% | 0.51 |
| Income (Percentile) |  |  |  |
| 76th-100^th^ | 32.7% | 29.4% | 0.35 |
| 51st-75^th^ | 24.1% | 23.4% | 0.78 |
| 26th-50^th^ | 23.7% | 26.0% | 0.35 |
| 0th-25^th^ | 18.1% | 19.8% | 0.45 |
| Payer Status |  |  |  |
| Private | 35.2% | 39.2% | 0.18 |
| Medicare | 50.8% | 51.2% | 0.89 |
| Medicaid | 9.6% | 6.3% | **0.033** |
| Other Payer | 4.2% | 3.0% | 0.27 |
| **Hospital Characteristics** |  |  |  |
| Region |  |  |  |
| Northeast | 34.6% | 33.9% | 0.87 |
| Midwest | 17.0% | 16.8% | 0.95 |
| South | 25.7% | 33.0% | 0.052 |
| West | 22.8% | 16.4% | **0.038** |
| Teaching Status |  |  |  |
| Rural | 1.3% | 1.6% | 0.52 |
| Urban Non-Teaching | 9.1% | 10.6% | 0.45 |
| Urban Teaching | 89.5% | 87.8% | 0.42 |
| Gastrectomy Volume (Percentile) |  |  |  |
| 0th-33rd | 10.1% | 9.4% | 0.68 |
| 34th-66th | 10.3% | 11.2% | 0.63 |
| 67th-100th | 79.5% | 79.4% | 0.94 |

Supplemental Table 3: Perioperative outcomes stratified by laparoscopic and robotic approach.

| **Parameter** | **Laparoscopic (4,698)** | **Robotic (2,544)** | **P-Value** |
| --- | --- | --- | --- |
| Mortality | 1.4% | 1.4% | 0.99 |
| **Complications** |  |  |  |
| Cardiac | 1.1% | 1.4% | 0.70 |
| Thrombotic | 2.3% | 1.9% | 0.71 |
| Respiratory | 17.8% | 19.3% | 0.45 |
| Gastrointestinal | 4.6% | 6.3% | 0.16 |
| Infectious | 10.9% | 8.5% | 0.15 |
| Acute Kidney Injury | 0.5% | 1.0% | 0.26 |
| Non-Routine Discharge | 45.7% | 50.5% | 0.13 |
| LOS (days, mean±SD) | 10.0±10.4 | 9.6±8.3 | 0.51 |
| Cost ($1,000, mean±SD) | 41.4±43.1 | 43.9±39.9 | 0.30 |
